# Deep Chest Wall Abscess Involving the Pectoralis Muscle Caused by Actinomyces turicensis and Bacteroides ovatus Mimicking Necrotizing Soft Tissue Infection

**DOI:** 10.7759/cureus.115819

**Published:** 2026-09-05

**Authors:** Jordan De Guzman, Martha Flores, Carolina Karuppiah, Alireza Izadian Bidgoli, Ehssan Zare

**Affiliations:** 1 Academic Department, American University of the Caribbean School of Medicine, Cupecoy, SXM; 2 Surgery Department, Henry Ford Providence, Southfield, USA

**Keywords:** bacterial pyomyositis, debridement, excisional debridement, necrotizing fasciitis, tropical infections

## Abstract

Deep chest wall abscesses may be difficult to distinguish from primary pyomyositis and necrotizing soft-tissue infection because of overlapping clinical and radiographic findings. Although soft-tissue gas raises concern for necrotizing infection, it is not independently diagnostic and may reflect anaerobic bacterial activity, communication with the skin, or prior instrumentation. Infections of the pectoral region involving uncommon polymicrobial organisms are rarely reported.

A 61-year-old man with prediabetes, hypertension, chronic kidney disease, and a prior cerebrovascular accident presented with a progressively worsening left anterior chest wall lesion after incomplete outpatient incision and drainage and a partial course of cephalexin. Computed tomography demonstrated a 7.0 × 4.1 × 2.2 cm gas-containing collection within the left upper anterior chest wall that abutted the pectoralis musculature, with associated muscular edema. These findings raised concern for necrotizing soft-tissue infection and prompted broad-spectrum antimicrobial therapy and urgent surgical evaluation. Initial bedside incision and drainage yielded copious purulent material. Persistent induration and loculations required repeat exploration, creation of a second incision, and placement of a Penrose drain. Cultures of material obtained directly from the abscess cavity grew *Actinomyces turicensis* and *Bacteroides ovatus*. Antimicrobial therapy was subsequently changed to intravenous ampicillin-sulbactam followed by oral amoxicillin-clavulanate. The patient improved following additional source control and culture-directed therapy, without evidence of fascial necrosis or a need for extensive debridement. At two-week follow-up, purulent drainage had resolved, and the Penrose drain was removed without complication.

This case describes an unusual gas-containing deep chest wall abscess involving the pectoral region with secondary muscular inflammation. It demonstrates the importance of distinguishing primary intramuscular infection from contiguous involvement by an adjacent abscess and recognizing that soft-tissue gas alone does not establish necrotizing infection. Prompt surgical assessment, adequate source control, appropriately obtained cultures, and integration of clinical, imaging, operative, and microbiologic findings are essential to guide management.

## Introduction

Pyomyositis is a bacterial infection of skeletal muscle characterized by muscle inflammation that may progress to intramuscular suppuration and abscess formation. Historically regarded as a tropical disease, pyomyositis is increasingly recognized in temperate regions, particularly among patients with underlying conditions such as diabetes mellitus, chronic kidney disease, and immunocompromising disorders [1,2]. *Staphylococcus aureus* remains the predominant causative organism, whereas polymicrobial and anaerobic infections are relatively uncommon. The pelvic girdle and lower-extremity muscle groups are most frequently affected, while involvement of the pectoral musculature is rare [1-3].

Primary pyomyositis should be distinguished from secondary muscular involvement caused by contiguous extension of a cutaneous, subcutaneous, or other adjacent infection. A diagnosis of pyomyositis is generally supported by imaging demonstrating inflammation or an abscess centered within skeletal muscle, direct drainage of intramuscular purulence, or operative confirmation of infected muscle. In contrast, muscular edema adjacent to a superficial or deep soft tissue abscess may represent reactive inflammation or secondary extension rather than primary pyomyositis [1-3].

Distinguishing these entities from cellulitis and necrotizing soft tissue infection can be challenging because their clinical and radiographic features may overlap [4,5]. In the present case, the infection began as a superficial boil that spontaneously ruptured and underwent outpatient incision and drainage before hospital presentation. Subsequent imaging demonstrated a gas-containing deep chest wall collection abutting the pectoralis musculature with associated muscular edema, but did not conclusively demonstrate an abscess centered within the muscle. We therefore describe this case as a deep chest wall abscess with pectoralis muscle involvement associated with the isolation of *Actinomyces turicensis* and *Bacteroides ovatus*. The case is notable for its unusual polymicrobial microbiology, the diagnostic concern created by intralesional gas, and the difficulty of distinguishing secondary muscular involvement from primary pyomyositis and necrotizing soft tissue infection.

## Case presentation

A 61-year-old male with a history of prediabetes, hypertension, hyperlipidemia, stage 3A chronic kidney disease, tobacco use disorder, and prior cerebrovascular accident presented to the emergency department with a progressively worsening left chest wall lesion. Approximately two weeks prior to presentation, he noticed a small boil over the superior aspect of his left chest wall without preceding trauma, insect bite, or skin manipulation. The lesion subsequently ruptured spontaneously, with purulent drainage.

He was subsequently evaluated by his primary care physician, where incision and drainage were performed. Despite the procedure, he reported persistent fullness and intermittent drainage, suggesting incomplete source control. He was prescribed cephalexin but completed only a partial course. Over the following days, the lesion progressively enlarged and became increasingly painful, accompanied by chills, fatigue, and decreased appetite, ultimately prompting presentation to the emergency department three days after his initial primary care visit.

On arrival, he was hemodynamically stable but tachycardic (109 beats per minute). Physical examination demonstrated a large area of induration and erythema over the superior left anterior chest wall overlying the pectoral region (Figure 1). Laboratory evaluation (Table 1) revealed leukocytosis and mild hyponatremia, with a normal lactate level. Although the lesion initially appeared consistent with a superficial soft tissue infection, contrast-enhanced computed tomography of the thorax revealed a 7.0 × 4.1 × 2.2 cm gas-containing fluid collection within the left upper anterior chest wall extending toward the clavicle. The collection abutted the left pectoralis musculature, with associated muscular edema and surrounding inflammatory fat stranding; however, a discrete intramuscular collection was not definitively identified (Figure 2). These findings raised concern for a deep chest wall infection, including a possible necrotizing soft tissue infection. Concern for necrotizing soft tissue infection was further heightened by a concomitant Laboratory Risk Indicator for Necrotizing Fasciitis (LRINEC) score of 6 [6].

**Figure 1:**
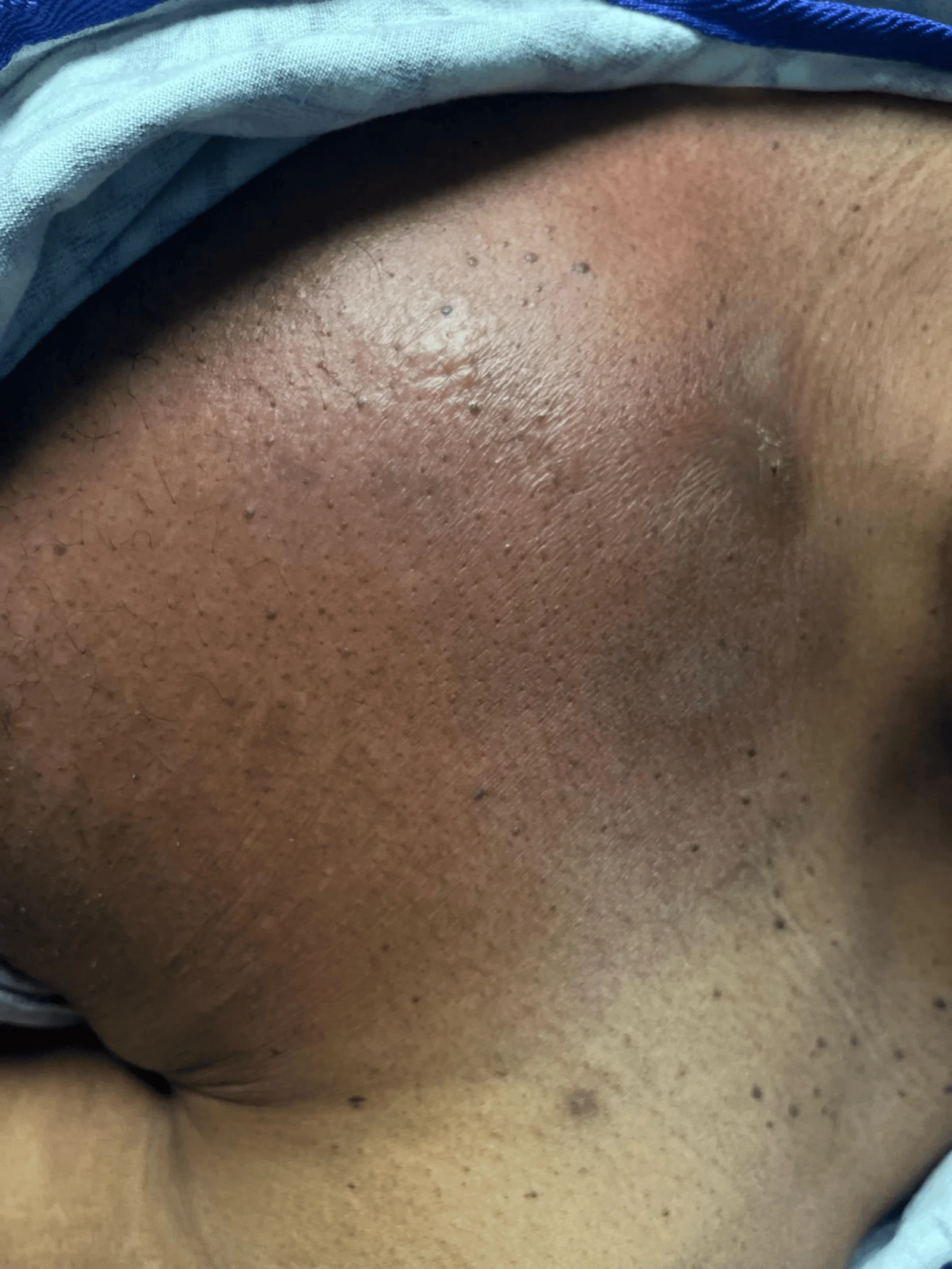
Initial Clinical Presentation of Left Anterior Chest Wall Lesion Clinical photograph obtained on presentation demonstrating a large area of erythema, induration, and soft tissue swelling involving the superior left anterior chest wall overlying the pectoral region.

**Table 1:** Laboratory Values on Arrival eGFR: Estimated Glomerular Filtration Rate; CRP: C-Reactive Protein; ESR: Erythrocyte Sedimentation Rate

Laboratory Test	Result	Reference Range	Units
White Blood Cell Count	14.7	4.0–11.0	×10³/µL
Hemoglobin	15	13.5–17.5	g/dL
Mean Corpuscular Volume	78.1	80–100	fL
Platelet Count	191	150–400	×10³/µL
Sodium	132	135–145	mmol/L
Potassium	3.6	3.5–5.0	mmol/L
Bicarbonate (CO₂)	19	22–29	mmol/L
Creatinine	1.4	0.7–1.3	mg/dL
eGFR	58	>60	mL/min/1.73 m²
Glucose	130	70–99	mg/dL
Phosphorus	1.9	2.5–4.5	mg/dL
Magnesium	1.7	1.7–2.2	mEq/L
Lactic Acid	0.9	0.5–2.0	mmol/L
CRP	197	<10	mg/L
ESR	47	0–20	mm/hr
Creatine Kinase	104	40–200	U/L

**Figure 2:**
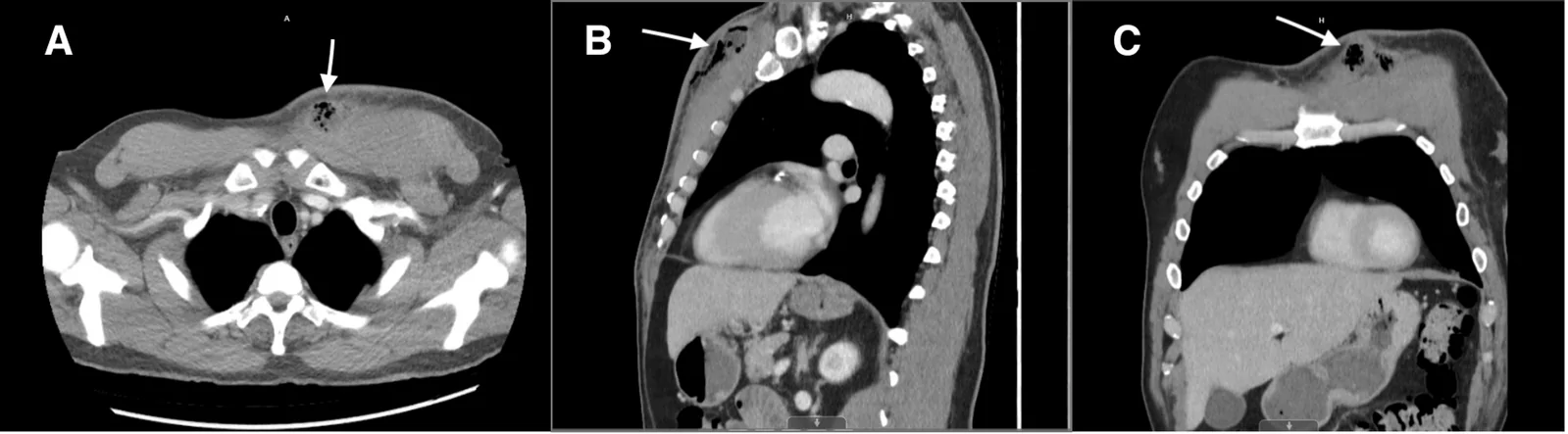
Contrast-Enhanced Computed Tomography of the Left Chest Wall Multiplanar contrast-enhanced computed tomography of the chest demonstrating the left anterior chest wall infection. (A) Axial view showing a focal fluid collection within the left upper anterior chest wall containing internal foci of gas (arrow). (B) Sagittal view demonstrating superior extension of the collection toward the clavicle with involvement of the pectoral musculature (arrow). (C) Coronal view confirming the localized gas-containing collection within the left anterior chest wall (arrow).

Given these findings, broad-spectrum intravenous antibiotics were initiated with vancomycin and cefepime, and general surgery was consulted. Bedside incision and drainage were performed, yielding copious amounts of purulent material. Tissue and purulent material samples were taken for Gram stain and aerobic and anaerobic cultures. During the procedure, multiple inward-facing hairs were noted within the lesion, suggesting a possible follicular origin of infection. The abscess cavity was explored with blunt dissection to disrupt loculations, irrigated, and packed, and samples were sent for microbiologic evaluation. Due to persistent concern for necrotizing soft tissue infection, linezolid and clindamycin were temporarily added to the initial vancomycin and cefepime regimen on hospital day 1 while cultures remained pending. Clindamycin was included for empiric toxin suppression.

Infectious disease consultation characterized the presentation as a deep soft tissue infection with possible early myositis involving the adjacent pectoralis musculature. However, the available imaging did not demonstrate a collection centered within the muscle, and the bedside drainage did not document intramuscular purulence or infected muscle. Therefore, primary pyomyositis could not be conclusively established. The patient’s initial superficial boil, spontaneous drainage, inward-growing hairs, and prior incision and drainage instead supported a possible follicular or cutaneous abscess with extension into the deeper chest wall tissues and secondary inflammatory involvement of the adjacent pectoralis muscle. The leukocyte counts subsequently decreased from 14.7 to 9.6 × 10³/μL. Creatine kinase remained within normal limits, and blood cultures showed no growth. With their guidance, vancomycin, cefepime, and clindamycin therapy was stopped, and only linezolid was continued.

Despite initial intervention, the patient continued to demonstrate persistent induration with evidence of loculated infection on day 3 of hospitalization. He underwent repeat bedside exploration with the creation of a second incision inferior to the original site. Blunt dissection was used to establish communication between the two cavities, allowing further evacuation of purulent material. A Penrose drain was placed through both incisions to facilitate ongoing drainage (Figure 3).

**Figure 3:**
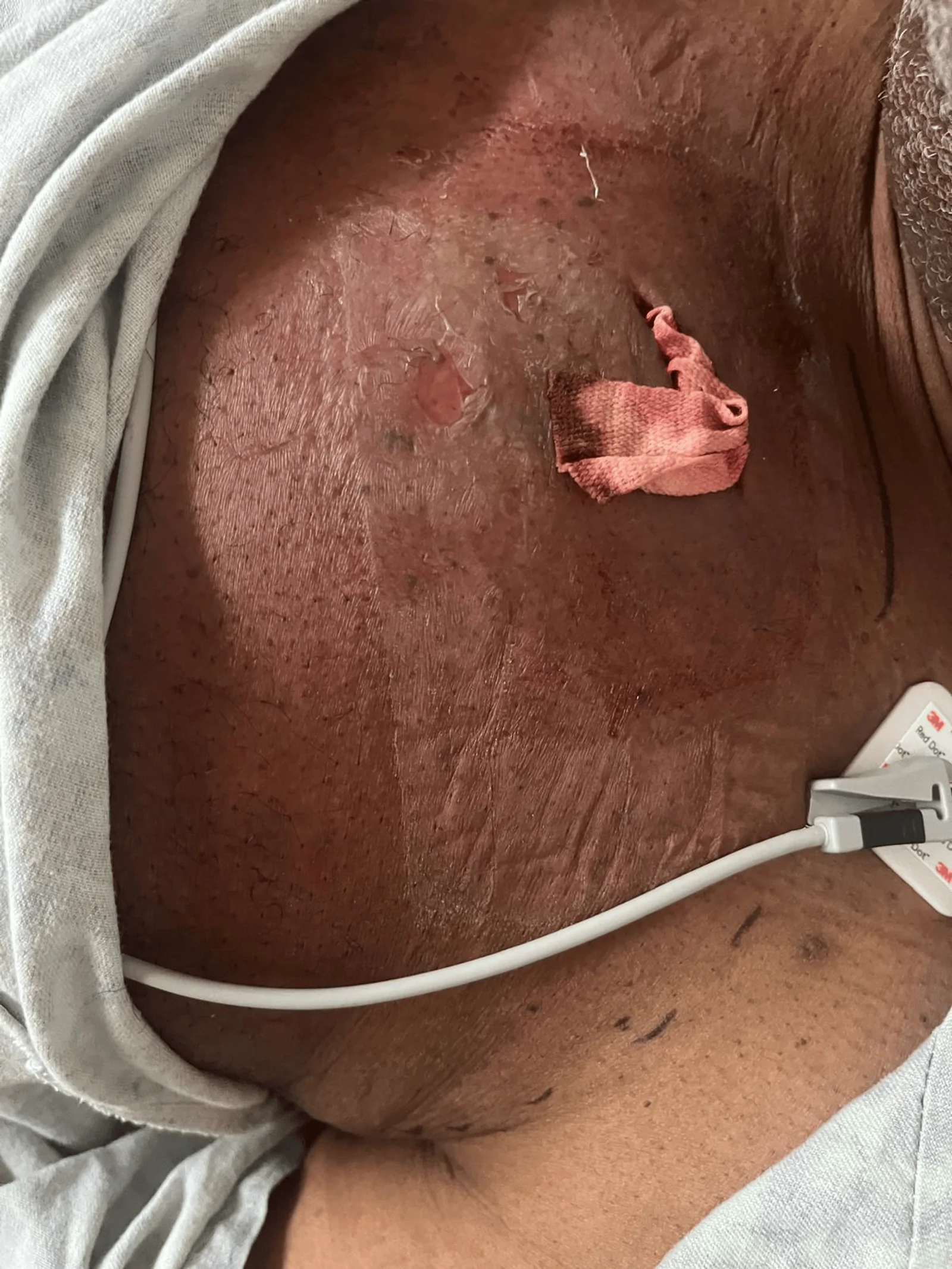
Clinical Appearance Following Repeat Incision and Drainage Clinical photograph obtained after repeat bedside incision and drainage demonstrating placement of a Penrose drain through the left anterior chest wall to facilitate continued drainage of the abscess cavity. Compared with the initial presentation (Figure 1), the surrounding erythema and induration had improved following repeat drainage and targeted antimicrobial therapy.

The wound cultures obtained from the initial incision and drainage on day 1 grew *Actinomyces turicensis*, while the anaerobic abscess culture grew *Bacteroides ovatus*. Blood cultures remained negative. Because both organisms were recovered from specimens obtained during drainage of the abscess cavity rather than from a superficial skin swab, they were considered clinically relevant components of the polymicrobial infection. Based on these findings, the Infectious Disease team transitioned antimicrobial therapy to intravenous ampicillin-sulbactam, followed by oral amoxicillin-clavulanate after adequate source control and clinical improvement. A prolonged course was recommended because of the isolation of *A. turicensis*.

The patient demonstrated interval clinical improvement in erythema and drainage, with the Penrose drain functioning appropriately and only mild residual purulent output. He remained hemodynamically stable and was discharged with close outpatient follow-up, and a 21-week course of amoxicillin-clavulanate antibiotics for continued management of his infection.

At outpatient follow-up approximately two weeks after discharge, the patient demonstrated continued clinical improvement, with resolution of purulent drainage and no evidence of residual infection. The Penrose drain was removed without complication, and the patient was encouraged to finish the course of antibiotics.

## Discussion

Pyomyositis, historically termed “tropical pyomyositis,” is increasingly recognized in temperate regions and most commonly affects the lower-extremity musculature. Its incidence in the United States increased more than threefold between 2002 and 2014, particularly among patients with underlying conditions such as diabetes mellitus and chronic kidney disease [1,2]. *Staphylococcus aureus* remains the predominant causative organism, accounting for approximately 90% of cases [1-3]. Although this patient had risk factors for a complicated soft tissue infection, including chronic kidney disease and prediabetes, the anatomical origin and extent of his infection created substantial diagnostic uncertainty.

Primary pyomyositis should be distinguished from reactive myositis or secondary muscular involvement caused by contiguous spread from an adjacent cutaneous or subcutaneous infection. Primary pyomyositis is generally supported by imaging demonstrating inflammation or an abscess centered within skeletal muscle, direct drainage of intramuscular purulence, or operative confirmation of infected muscle [2,3]. In this case, CT demonstrated a gas-containing anterior chest wall collection abutting the pectoralis musculature, with associated muscular edema, but did not definitively demonstrate a collection centered within the muscle. Furthermore, bedside drainage did not document intramuscular purulence or infected muscle. The preceding superficial boil, spontaneous rupture, inward-growing hairs, and outpatient incision and drainage support an alternative interpretation of a follicular or cutaneous abscess with extension into the deeper chest wall tissues and secondary inflammation of the adjacent pectoralis muscle. Although early pyomyositis was considered clinically, it could not be conclusively established.

Diagnostic uncertainty was present at hospital presentation because cellulitis, a deep chest wall abscess, secondary muscular involvement, and necrotizing soft tissue infection were considered. These conditions may share localized erythema, tenderness, swelling, fever, leukocytosis, and induration, while fluctuance may be absent when an abscess lies within deeper tissues [4,7]. In this patient, progression despite outpatient drainage and an incomplete course of oral antibiotics prompted imaging and evaluation for an inadequately drained or more extensive infection.

Distinguishing a non-necrotizing abscess from a necrotizing soft tissue infection is critical because suspected necrotizing infection requires urgent surgical assessment. Findings that may increase concern include rapid progression, pain disproportionate to examination, systemic toxicity, crepitus, fascial thickening, and fluid tracking along the deep fascia, although classic features are not always present [4,5]. Fluid tracking along the deep fascia on CT has been reported to have a specificity of 94% for necrotizing soft tissue infection [8]. This finding is distinct from soft tissue gas and should not be conflated with it. In the present case, the gas-containing collection appropriately raised concern for necrotizing infection, but the available imaging did not demonstrate definite fascial necrosis or deep fascial fluid tracking.

The intralesional gas could not be attributed definitively to a single mechanism. Possible explanations included anaerobic bacterial activity, residual air introduced during the preceding outpatient incision and drainage, or prior communication with the skin following spontaneous rupture. Although no widely patent cutaneous opening was apparent at hospital presentation, a previously patent or intermittently draining tract could not be excluded. Thus, the presence of gas heightened concern for necrotizing infection but was not independently diagnostic of fascial necrosis [4,5]. General surgery performed prompt bedside exploration and drainage, followed by repeat exploration for persistent loculations. Serial surgical assessments did not identify necrotic fascia or other findings requiring extensive debridement.

The patient’s LRINEC score of 6 met the original threshold for increased concern and contributed to urgent surgical evaluation. However, the score was largely driven by an elevated C-reactive protein level and mild hyponatremia. The LRINEC score has important sensitivity and specificity limitations and should not be used independently to confirm or exclude necrotizing infection [6]. Similarly, the patient’s hemodynamic stability and normal lactate level did not exclude an early necrotizing process. The decision not to perform extensive debridement was based on serial clinical and surgical assessments rather than the LRINEC score, lactate level, or hemodynamic stability alone.

Microbiologic evaluation demonstrated a polymicrobial infection. Purulent material was collected directly from within the abscess cavity during bedside incision and drainage rather than by superficial wound swab. Culture of this specimen grew *Actinomyces turicensis*, while anaerobic culture of material from the same abscess cavity yielded *Bacteroides ovatus*. Although​​​ *Actinomyces* species may be recovered as components of normal mucosal flora or polymicrobial specimens, isolation of *A. turicensis *directly from the abscess cavity, together with *B. ovatus*, supported their clinical relevance in this infection [9].

Incomplete outpatient drainage likely contributed to the persistence of infection. Cephalexin also provides limited anaerobic activity and does not reliably cover *Bacteroides *species [10]. Initial inpatient treatment consisted of empiric broad-spectrum therapy while necrotizing infection was evaluated, followed by modification as the surgical assessment and microbiologic results became available. Once *A. turicensis *and *B. ovatus *were identified, therapy was changed to ampicillin-sulbactam to provide beta-lactam and anaerobic coverage, followed by oral amoxicillin-clavulanate after adequate source control and clinical improvement.

Management of infections involving *Actinomyces *species generally includes adequate source control and beta-lactam antimicrobial therapy. *Actinomyces* ​​​​​species are generally susceptible to penicillins and ampicillin-sulbactam but are intrinsically resistant to metronidazole [11]. Although prolonged treatment courses have traditionally been recommended, shorter courses may be effective when adequate drainage or resection has been achieved [10,12]. In this patient, repeat drainage, Penrose drain placement, and culture-directed antimicrobial therapy resulted in clinical resolution without extensive debridement. At follow-up approximately two weeks after discharge, there was no residual purulence or evidence of ongoing infection, and the Penrose drain was removed without complication. This case emphasizes the importance of defining the anatomical extent of deep chest wall infections, obtaining appropriately collected cultures, and integrating imaging with serial surgical assessment when necrotizing soft tissue infection remains a concern.

## Conclusions

This case demonstrates the diagnostic challenge posed by a gas-containing deep chest wall abscess involving the pectoralis region. Although soft-tissue gas appropriately raises concern for necrotizing infection, it is not independently diagnostic and may result from anaerobic bacterial activity, communication with the skin, or prior instrumentation. Hemodynamic stability, a normal lactate level, and imaging findings alone cannot exclude necrotizing soft-tissue infection; prompt surgical evaluation remains essential. In this patient, drainage and serial surgical examinations showed no evidence of fascial necrosis, permitting treatment with source control and culture-directed antimicrobial therapy without extensive debridement. Recovery of *Actinomyces turicensis *and *Bacteroides ovatus *from the abscess cavity further demonstrates the value of appropriately obtained cultures in guiding treatment of atypical polymicrobial infections.
